# Early-Life Prevention of Cardiovascular–Kidney–Metabolic Syndrome: The DOHaD Perspective on Resveratrol and Short-Chain Fatty Acids

**DOI:** 10.3390/antiox14070851

**Published:** 2025-07-10

**Authors:** Chien-Ning Hsu, Ying-Jui Lin, Chih-Yao Hou, Yu-Wei Chen, You-Lin Tain

**Affiliations:** 1 Department of Pharmacy, Kaohsiung Chang Gung Memorial Hospital, Kaohsiung 833, Taiwan; cnhsu@cgmh.org.tw; 2 Department of Pharmacy, Kaohsiung Municipal Ta-Tung Hospital, Kaohsiung 801, Taiwan; 3 School of Pharmacy, Kaohsiung Medical University, Kaohsiung 807, Taiwan; 4 Division of Critical Care, Department of Pediatrics, Kaohsiung Chang Gung Memorial Hospital and Chang Gung University College of Medicine, Kaohsiung 833, Taiwan; rayray@adm.cgmh.org.tw; 5 Division of Cardiology, Department of Pediatrics, Kaohsiung Chang Gung Memorial Hospital and Chang Gung University College of Medicine, Kaohsiung 833, Taiwan; 6 Department of Respiratory Therapy, Kaohsiung Chang Gung Memorial Hospital and Chang Gung University College of Medicine, Kaohsiung 833, Taiwan; 7 Department of Early Childhood Care and Education, Cheng Shiu University, Kaohsiung 833, Taiwan; 8 Department of Seafood Science, National Kaohsiung University of Science and Technology, Kaohsiung 811, Taiwan; chihyaohou@nkust.edu.tw; 9 Department of Food Science and Biotechnology, National Chung Hsing University, Taichung 402, Taiwan; d112043001@mail.nchu.edu.tw; 10 Department of Pediatrics, Kaohsiung Chang Gung Memorial Hospital, Kaohsiung 833, Taiwan; 11 Department of Pediatrics, Kaohsiung Municipal Ta-Tung Hospital, Kaohsiung 801, Taiwan; 12 College of Medicine, Chang Gung University, Taoyuan 333, Taiwan

**Keywords:** kidney disease, cardiovascular disease, metabolic syndrome, oxidative stress, gut microbiota, resveratrol, short-chain fatty acids, developmental origins of health and disease (DOHaD), developmental programming

## Abstract

Cardiovascular–kidney–metabolic (CKM) syndrome underscores the interconnected biology of cardiovascular disease, kidney disease, and metabolic disorders such as obesity and type 2 diabetes. Although now recognized as a growing global health burden, accumulating preclinical evidence suggests that CKM syndrome may originate in early life—a concept rooted in the developmental origins of health and disease (DOHaD) framework. Animal studies have greatly enhanced our comprehension of these mechanisms, emphasizing the promise of early interventions that focus on antioxidants and gut microbiota modulation to mitigate the development of CKM conditions. Resveratrol, a natural antioxidant and prebiotic, alongside short-chain fatty acids (SCFAs), a postbiotic, have demonstrated the ability to modulate gut microbiota and oxidative stress in experimental models. Various resveratrol derivatives have also been engineered to improve bioavailability, though their effects remain largely confined to animal studies. This review synthesizes preclinical findings on the impact of perinatal oxidative stress and gut dysbiosis on CKM outcomes, critically examining the roles of resveratrol, SCFAs, and their derivatives in animal models. Finally, we highlight the significant translational gap between experimental research and clinical application, underscoring the need for human studies to validate these early-life intervention strategies.

## 1. Introduction

Cardiovascular–kidney–metabolic (CKM) syndrome is a developing clinical concept that acknowledges the complex interactions between cardiovascular disease, chronic kidney disease (CKD), and metabolic disorders like obesity, insulin resistance, and type 2 diabetes [1,2]. Rather than treating these conditions as isolated entities, CKM syndrome highlights their shared risk factors and common mechanistic pathways—including oxidative stress, endothelial dysfunction, inflammation, and gut microbiota dysbiosis—that often originate early in life [3,4,5]. This integrated framework promotes earlier identification of at-risk individuals and encourages holistic prevention strategies [6]. Growing evidence also supports the role of early-life exposures and nutritional interventions in shaping long-term CKM health, aligning closely with the principles of the developmental origins of health and disease (DOHaD) [6].

Trillions of microbes reside in the human gut, collectively forming the gut microbiota—a complex ecosystem that has coevolved with humans in a mutually beneficial relationship. Disruptions to this microbial balance, known as dysbiosis, have been increasingly linked to various CKM conditions, including obesity, type 2 diabetes, metabolic syndrome, CKD, hypertension, and CVD [7,8,9,10]. Emerging evidence suggests that adverse maternal environments and early-life exposures have a critical role in shaping the development of the offspring’s gut microbiome, with long-term implications for adult CKM health [11]. On the other hand, approaches aimed at modulating the gut microbiota—through the use of probiotics, prebiotics, and postbiotics—have demonstrated potential in enhancing metabolic programming and improving CKM-related outcomes in offspring [12,13]. Among these, resveratrol and short-chain fatty acids (SCFAs) stand out for their ability to modulate gut microbiota composition and function through prebiotic-like and postbiotic mechanisms, respectively.

Resveratrol (trans-3,5,4′-trihydroxystilbene) is one of the most studied polyphenols in the stilbene family [14]. Found in a variety of plants, it features a C6–C2–C6 backbone and three hydroxyl groups, contributing to its potent antioxidant properties [15,16,17,18]. Although not a classical prebiotic, resveratrol exerts prebiotic-like effects by modulating gut microbial communities and their metabolic outputs. Despite its therapeutic potential, clinical translation is challenged by resveratrol’s rapid metabolism and poor bioavailability [19]. Consequently, enhancing its bioavailability has become a key focus of ongoing research [20].

SCFAs, including acetic (C2), propionic (C3), and butyric (C4) acids, are key microbial metabolites produced through the fermentation of dietary fibers and polysaccharides [21]. They are known to protect against oxidative stress and regulate diverse metabolic and inflammatory pathways involved in CKM pathogenesis [22,23]. Recent studies suggest that both resveratrol and SCFAs may exert synergistic effects on gut microbiota modulation, offering therapeutic potential in the prevention and management of CKM conditions [24]. Moreover, novel approaches such as the synthesis of resveratrol–SCFA esters are being explored to enhance bioactivity and improve outcomes in CKM-related diseases [25,26].

This narrative review aims to provide an updated synthesis of current research and highlight recent advances in identifying resveratrol, SCFAs, and their derivatives as promising agents for the prevention of CKM syndrome, with particular focus on their roles in modulating gut microbiota and oxidative stress.

## 2. Material and Methods

To ensure a thorough and up-to-date synthesis of relevant research, we performed a comprehensive literature search covering studies published in English from January 2000 to April 2025. The search strategy encompassed both experimental (animal) and clinical studies, with primary sources retrieved from major scientific databases, including MEDLINE, the Cochrane Library, and Embase. Priority was given to full-text English-language publications. Keywords and subject headings were selected to capture a wide range of concepts related to oxidative stress, gut microbiota, resveratrol, SCFAs, DOHaD, and CKM syndrome. Search terms included, but were not limited to: “oxidative stress”, “nitric oxide”, “reactive oxygen species”, “antioxidants”, “gut microbiota”, “microbiome”, “short-chain fatty acid”, “acetate”, “propionate”, “butyrate”, “obesity”, “diabetes”, “chronic kidney disease”, “metabolic syndrome”, “cardiovascular disease”, “hypertension”, “dyslipidemia”, “insulin resistance”, “hyperlipidemia”, “hyperglycemia”, “hepatic steatosis”, “atherosclerosis”, “heart failure”, “developmental programming”, “reprogramming”, “DOHaD”, “offspring”, “progeny”, “maternal”, “pregnancy”, and “lactation.” Reference lists of selected studies were also manually screened to identify additional relevant literature not captured in the initial database searches.

## 3. Oxidative Stress and Developmental Programming in CKM Syndrome

### 3.1. Human Evidence

Epidemiological studies from famine cohorts [27,28,29] and twin research [30,31] support the concept that adverse intrauterine environments—such as maternal malnutrition, obesity, diabetes, and stress—are linked to low birth weight and a higher risk of CKM conditions later in life, such as obesity, hypertension, dyslipidemia, insulin resistance, and CVD [32,33,34,35]. These findings align with the DOHaD hypothesis but offer limited insight into the molecular mechanisms, particularly oxidative stress, that mediate long-term health outcomes [36].

### 3.2. Oxidative Stress in Developmental Programming

Animal models have been instrumental in revealing how early-life oxidative stress contributes to CKMS. Maternal insults—like undernutrition [37], diabetes [38], toxin exposure [39], and pregnancy complication [40]—can increase reactive oxygen species (ROS) in utero, disrupting redox balance and nitric oxide (NO) signaling during critical stages of fetal development. This redox imbalance impairs cardiovascular, kidney, and metabolic programming, predisposing offspring to various CKM condition, including hypertension, insulin resistance, and obesity.

### 3.3. Key Mechanisms

Maintaining the balance between ROS and antioxidants during pregnancy is crucial for optimal fetal development [41]. Oxidative stress, arising from this imbalance, plays a central role in CKM programming in compromised pregnancies [42]. Key mechanisms include the upregulation of ROS-generating enzymes, diminished antioxidant defenses (e.g., catalase and superoxide dismutase), and disruption of NO pathways via elevated levels of asymmetric dimethylarginine (ADMA, an NO synthase inhibitor) [36]. Notably, accumulating evidence from animal models suggests that redox disturbances and their downstream effects are often sex-specific, with male offspring typically exhibiting greater oxidative damage, heightened inflammation, and more severe CKM phenotypes compared to females. These sex differences have been reported in models involving maternal nicotine exposure [43], prenatal glucocorticoid treatment [44], ethanol consumption [45], and litter-size reduction [46].

Biomarkers such as 3-nitrotyrosine (3-NT) [37], F2-isoprostanes [40], malondialdehyde [43], 8-hydroxydeoxyguanosine (8-OHdG) [47], and 4-hydroxynonenal (4-HNE) [48] further confirm oxidative damage in affected tissues, including the kidneys, liver, and vasculature. Animal models that replicate multiple CKM-related phenotypes in a sex-dependent manner provide valuable translational insight and reinforce oxidative stress as a critical target for early intervention. Elucidating these sex-specific redox mechanisms may refine reprogramming strategies aimed at reducing the long-term burden of CKM syndrome in both sexes.

## 4. Gut Microbiota and Developmental Programming in CKM Syndrome

### 4.1. Early-Life Microbiota and Developmental Programming

Emerging evidence identifies the gut microbiota as a key modulator in DOHaD-related disorders, including the pathogenesis of CKM syndrome [11,12]. Neonatal gut colonization begins at birth [49,50], with the microbiome gradually diversifying and achieving an adult-like composition by around two years of age. The composition of the infant gut microbiota is strongly influenced by maternal factors such as diet, obesity, antibiotic exposure, and metabolic status [51]. Disruptions during this critical developmental window can lead to long-lasting alterations in host physiology via microbiota-mediated programming of metabolic, cardiovascular, and kidney systems [52,53].

### 4.2. Mechanistic Contributions of Gut Microbiota Dysbiosis

Early-life gut dysbiosis contributes to CKM syndrome through several interrelated mechanisms. First, reduced production of microbial-derived SCFAs compromises gut barrier integrity, insulin sensitivity, and anti-inflammatory responses, all of which are associated with CKM syndrome [54]. Second, dysbiosis disrupts tight junction proteins, facilitating the translocation of lipopolysaccharides (LPS) into systemic circulation. This triggers low-grade endotoxemia and chronic inflammation [55]. LPS-induced activation of the TLR4–NFκB signaling pathway further enhances ROS production and pro-inflammatory cytokine expression, exacerbating oxidative damage [56].

Third, inflammatory signaling and microbial metabolites interfere with endothelial nitric oxide synthase (eNOS) activity, resulting in reduced NO bioavailability, endothelial dysfunction, and hypertension [57]. Fourth, dysbiotic microbiota enhance microbial metabolism of tryptophan, increasing production of uremic toxins such as indoxyl sulfate and p-cresyl sulfate—both implicated in kidney fibrosis, oxidative stress, and CKD progression [52]. Lastly, elevated levels of trimethylamine N-oxide (TMAO) are associated not only with CVD mortality [58] but also with increased risks of CKD, type 2 diabetes, insulin resistance, and metabolic dysfunction-associated fatty liver disease [59].

### 4.3. Experimental Evidence

Rodent models involving maternal high-fat diets [60], high-fructose intake [61], or CKD [62] demonstrate that offspring exposed to dysbiotic microbial environments are more likely to develop CKM syndrome. The reversal or attenuation of these phenotypes through interventions such as probiotics, prebiotics, postbiotics, or fecal microbiota transplantation (FMT) supports a causal role for the gut microbiota in developmental programming [60,61,62,63]. These results highlight the therapeutic value of focusing on the gut’s interaction with both the kidneys and metabolic processes. Modulating maternal or neonatal microbiota may offer novel strategies for the early prevention of CKM syndrome through microbiome-targeted reprogramming.

### 4.4. Translational Relevance and Future Directions

Although preclinical models have elucidated key mechanisms linking early-life dysbiosis to CKM syndrome, translation to human populations remains limited. Longitudinal cohort studies are beginning to reveal how microbiome trajectories across infancy, childhood, and adolescence are shaped by early exposures—including mode of delivery, antibiotic use, diet, and maternal metabolic health—and how these patterns correlate with CKM conditions later in life [11,50,51]. Sex-specific and exposure-dependent differences in microbiota–host interactions further complicate this landscape, underscoring the need for integrative, multi-omics approaches. Future research should focus on linking microbiota signatures with functional outcomes and biomarkers, ultimately enabling targeted interventions and precision prevention strategies tailored to at-risk developmental windows.

## 5. Reprogramming Approach for Preventing CKM Syndrome

Reprogramming strategies aim to prevent or reverse the long-term consequences of developmental programming before clinical symptoms of CKM syndrome manifest. These interventions are typically applied during critical windows of development—namely, gestation and early postnatal life. Strategies include nutritional modulation, pharmacological agents, physical activity, and microbiota-targeted therapies. Given the pivotal roles of oxidative stress and gut dysbiosis in CKM pathogenesis, early-life interventions targeting these mechanisms are urgently needed in animal models before translation to human applications.

To date, a range of reprogramming approaches—including antioxidants and microbiome-directed therapies—have been investigated to mitigate specific components of CKM syndrome, such as hypertension [64], kidney disease [65], metabolic disorders [36,66], and CVD [67]. This review focuses on early-life interventions using resveratrol, SCFAs, and their derivatives during gestation and lactation as preventive strategies against offspring CKM syndrome. Each will be discussed in detail below.

### 5.1. Resveratrol

#### 5.1.1. Synthesis and Metabolism

Resveratrol is a plant-derived polyphenol synthesized in response to environmental stressors like pathogen attacks, mechanical damage, or UV radiation [68]. It occurs in two isomeric forms: *trans*- and *cis*-resveratrol. The biosynthesis involves the conversion of glucose to 4-coumaroyl-CoA, which then combines with malonyl-CoA via stilbene synthase to form trans-resveratrol. While *trans*-resveratrol is found predominantly in grapes, *cis*-resveratrol and its glucosides are present in wine, resulting partly from vinification processes that convert *trans*- to *cis*-resveratrol. *Trans*-resveratrol is stable in the absence of light but degrades at high pH, whereas *cis*-resveratrol is stable only in neutral conditions [69].

Following oral intake, approximately 70% of resveratrol is absorbed through passive diffusion or via intestinal transporters. However, it undergoes extensive first-pass metabolism in the liver, primarily via sulfation and glucuronidation, leading to very low levels of free resveratrol in circulation [70]. The major circulating forms are sulfate (e.g., trans-resveratrol-3,4′-disulfate) and glucuronide conjugates (e.g., trans-resveratrol-3-glucuronide), with a half-life of 130–180 min. Additional metabolites, including piceatannol and dihydroresveratrol, as well as gut microbiota-derived compounds, contribute to its overall bioactivity [71,72]. Inter-individual variation in absorption—ranging from 20–70% in humans and 15–50% in rats—is influenced by dosage, administration route, and gut microbiome composition [73,74].

#### 5.1.2. Interaction with Microbiota

Resveratrol exhibits bidirectional interactions with the gut microbiota. On the one hand, it modulates microbial composition and diversity; on the other, gut bacteria metabolize resveratrol into bioactive derivatives [75]. Its beneficial effects on obesity, diabetes, and atherosclerosis are partly attributed to its prebiotic action, which alters the gut microbiome and associated metabolite profiles [76]. For example, *Slackia equolifaciens* and *Adlercreutzia equolifaciens* have been identified as key bacteria responsible for converting resveratrol into dihydroresveratrol [77]. Additional microbial metabolites such as lunularin and 3,4-dihydroxy-trans-stilbene further demonstrate the role of gut microbiota in resveratrol metabolism.

#### 5.1.3. Anti-Oxidant and Other Biological Functions

Resveratrol is a versatile compound with a wide range of biological effects. It improves endothelial function, provides antioxidant and anti-inflammatory benefits, inhibits platelet aggregation, and exhibits anti-obesity, anticancer, and anti-atherosclerotic activities [72,76]. Additionally, it functions as an antagonist to the aryl hydrocarbon receptor (AhR) and enhances NO bioavailability [78,79,80]. Resveratrol influences numerous molecular pathways, such as nuclear factor-kappa B (NF-κB), nuclear factor-kappa B (NF-κB), silent information regulator-1 (SIRT-1), estrogen receptor α (ERα), CREB-binding protein, mammalian target of rapamycin (mTOR), adenosine monophosphate-activated protein kinase (AMPK), peroxisome proliferator-activated receptor (PPAR), activating transcription factor 2 (ATF2), and nuclear factor (erythroid-derived2)-like 2 (Nrf2), among others [80]. These diverse pathways contribute to its therapeutic promise.

#### 5.1.4. Prevention of CKM Syndrome by Resveratrol

Table 1 summarizes experimental studies evaluating the efficacy of maternal resveratrol supplementation during gestation and lactation in preventing offspring CKM syndrome [61,81,82,83,84,85,86,87,88,89,90,91,92].

Various environmental insults—including maternal nutritional imbalances [61,81,85,86], disease states [82,83,91], and chemical exposures [84,88,89]—have been modeled. In most studies, resveratrol was administered via drinking water at 50 mg/L [61,81,82,83,84,85,86,87,88,89], with some utilizing dietary supplementation (2–4 g/kg of chow) [91,92]. While limited research exists in large animals, two non-human primate studies suggest maternal resveratrol can improve maternal and placental function and benefit fetal liver development in mothers consuming Western-style diets [93,94]. However, the long-term impact on CKM outcomes remains unknown.

Evidence from rodent models supports the antioxidant role of resveratrol. For example, maternal resveratrol reduced renal 8-OHdG levels—a marker of oxidative DNA damage—and protected against offspring hypertension induced by maternal CKD [82]. It also mitigated hypertension programmed by maternal exposure to 2,3,7,8-tetrachlorodibenzo-p-dioxin (TCDD) and dexamethasone [88], as well as bisphenol A (BPA) combined with a high-fat diet [89].

Oxidative stress-related CKM programming disrupts NO signaling [95]. Resveratrol lowers ADMA and restores NO, offering vascular protection in several models [82,83,84,85,86,87]. Additionally, maternal resveratrol supplementation reshaped gut microbiota in offspring. In a CKD model, it increased beneficial genera like *Bifidobacterium* and *Lactobacillus* and enhanced microbial diversity [82]. In high-fructose diet models, similar shifts were observed alongside lowered BP [61]. In the L-NAME + high-fat diet model, resveratrol reduced the *Firmicutes*-to-*Bacteroidetes* ratio—an established microbial marker linked to hypertension and kidney disease [87]. These results suggest that maternal resveratrol may act as a prebiotic agent to reprogram the gut microbiome and prevent CKM syndrome.

Although resveratrol is generally considered safe at low to moderate doses in humans [96,97], high doses in animal studies have been associated with hepatotoxicity and gastrointestinal disturbances [98]. At high concentrations, resveratrol may exert pro-oxidant effects, generating ROS rather than acting as an antioxidant as observed at lower doses [99]. Translational application to human studies remains necessary, particularly with careful consideration of dosing, as human-equivalent doses derived from animal studies vary considerably [100].

### 5.2. Short-Chain Fatty Acids

#### 5.2.1. SCFAs and Fetal Programming

Short-chain fatty acids (SCFAs), mainly produced by gut microbiota, are involved in regulating fetal development [101]. These versatile compounds serve as energy sources, histone deacetylase (HDAC) inhibitors, and signaling molecules, allowing SCFAs to influence both fetal growth and metabolic processes [16]. Maternal SCFAs can affect the fetal SCFA levels via placental transfer, thereby impacting the metabolism of the developing offspring through the activation of widely distributed SCFA receptors.

SCFAs play distinct roles in maternal glucose and lipid metabolism. Acetate and butyrate are inversely associated with impaired glucose tolerance in a concentration-dependent manner. Butyrate is inversely associated with systemic inflammation, BMI, and gestational weight gain, while showing a positive correlation with total cholesterol and LDL levels in gestational diabetes mellitus (GDM) [102]. Acetate may reduce hepatic triglyceride accumulation and glucose dysregulation under high-fructose conditions [103].

Adiponectin and leptin, key adipokines, regulate insulin sensitivity and energy balance. An increased leptin-to-adiponectin ratio is a known feature of diabetes and obesity [104,105,106]. In pregnancy, serum acetate is linked to weight gain and adiponectin, while propionate inversely correlates with maternal leptin and infant length and weight.

SCFA signaling is often disrupted in metabolic disorders. G-protein–coupled receptors (GPRs), such as GPR41 and GPR43, mediate SCFA effects. During pregnancy, islet GPR43 expression increases, possibly supporting insulin resistance and glucose homeostasis [107]. Loss of GPR43 reduces cecal SCFAs, worsens antibiotic-induced gut dysbiosis, and leads to glucose intolerance [108]. GDM placentas show lower GPR41/GPR43 expression, but upregulation of downstream targets HDAC4, HDAC8, and HDAC9, suggesting impaired SCFA signaling and epigenetic regulation [102].

Low SCFA levels may activate lipid transporter CD36 by suppressing the HDAC3–H3K27ac–PPAR-γ axis, disrupting fatty acid transport and insulin sensitivity [109]. Conversely, SCFA supplementation or high-fiber diets improve insulin-to-glucagon ratios, β-cell mass, glucose tolerance, and insulin sensitivity in GDM models by engaging GPR pathways [110,111].

Although studies working on SCFAs and CKM programming are limited, we can gain insights from dysregulated SCFA-related signaling molecules and the response of the offspring phenotypes to maternal insults. Maternal high-fat diet leads to obesity and hypertension in male offspring [60], effects that can be mitigated by inulin, probiotics, or garlic oil through modulation of gut microbiota—particularly by lowering the *Firmicutes*-to-*Bacteroidetes* ratio, restoring fecal SCFAs like acetate and propionate, and regulating the renin–angiotensin system (RAS), NO, and hydrogen sulfide (H_2_S) pathways [112]. While SCFAs show potential in supporting fetal cardiovascular development and preventing programming-induced hypertension, their effects may vary depending on SCFA receptor affinities and proportions, as seen in TCDD-induced models with altered microbiota and RAS activation. Notably, acetate exhibits both vasoconstrictive and vasodilatory actions via Olfr78 and GPR41, highlighting the complexity of SCFA-mediated BP regulation [113].

#### 5.2.2. Prevention of CKM Syndrome by SCFAs

Several therapeutic approaches focus on modulating SCFAs, such as SCFA supplementation, providing SCFA precursors (e.g., fiber), or using probiotics and other drugs that influence related signaling pathways [101]. Table 2 highlights key studies that examined the direct effects of SCFA supplementation on animal models of CKM syndrome [114,115,116,117,118].

To date, five studies have demonstrated the reprogramming potential of SCFA supplementation—specifically acetate, butyrate, and propionate—in preventing programmed hypertension across various maternal insult models, including high-fructose intake [114,117], minocycline exposure [115], a tryptophan-free diet [116], and CKD [119].

In a maternal high-fructose diet model, acetate supplementation conferred protection against offspring hypertension by reducing plasma trimethylamine (TMA) levels, lowering the TMA-to-TMAO ratio, and increasing renal expression of SCFA receptors [114]. In the minocycline exposure model, maternal acetate supplementation mitigated offspring hypertension and was associated with an increased abundance of probiotic genera *Roseburia* and *Bifidobacterium* [115]. Perinatal butyrate supplementation prevented hypertension in offspring of mothers fed a tryptophan-free diet, with observed benefits linked to changes in gut microbiota composition, enhanced renal SCFA receptor expression, and restoration of RAS balance [116]. Similarly, perinatal propionate supplementation protected against maternal CKD-induced hypertension through modulation of gut microbiota, enrichment of propionate-producing bacteria, elevated plasma propionate levels, and increased renal SCFA receptor expression [118].

Only one study has directly compared the effects of perinatal butyrate versus propionate supplementation in a maternal high-fructose diet model. While both treatments elevated plasma levels of propionic acid, isobutyric acid, and valeric acid in adult offspring, butyrate exerted a greater impact on TMAO metabolism and NO pathways, whereas propionate primarily influenced gut microbiota composition [117].

Importantly, SCFAs exhibit differential vascular effects that may influence their antihypertensive efficacy. Acetate and butyrate can promote vasodilation by activating endothelial GPR41 and GPR43 [113], enhancing NO bioavailability, and reducing oxidative stress. Conversely, acetate may also activate Olfr78 receptors, particularly in the kidneys, leading to vasoconstriction and renin release [113]. These context- and receptor-dependent actions underscore the complexity of SCFA signaling and highlight the need for careful consideration of dose, receptor expression patterns, and host metabolic status when designing SCFA-based interventions.

Despite these promising findings, the protective effects of perinatal SCFA supplementation on CKM outcomes beyond hypertension remain largely unexplored. Notably, one study reported that maternal sodium butyrate supplementation enhanced lipolysis in adipose tissue but also led to hepatic lipid accumulation in weaning-age offspring [119], raising concerns about potential adverse effects. These observations underscore the need for further investigation into the broader therapeutic potential and safety of SCFAs as reprogramming agents across diverse CKM outcomes, as well as for a deeper understanding of their underlying mechanisms.

### 5.3. Resveratrol Derivatives

As noted earlier, the low bioavailability of resveratrol remains a significant obstacle to its clinical translation [73,120]. To address this, various strategies have been explored, including the synthesis of resveratrol derivatives with improved pharmacokinetics and biological activity [121]. These approaches include structural modifications [25], advanced delivery systems such as nanoparticles and liposomes [122,123,124], and enhancing bioaccessibility, which is significantly influenced by gut microbiota metabolism and intestinal absorption [125,126]. Resveratrol derivatives—structurally modified forms of the parent compound—have been designed to overcome limitations in stability, absorption, and metabolic reactivity. Common modifications include esterification with short- or long-chain fatty acids, glycosylation, methylation, and nanoformulation. These derivatives have shown enhanced antioxidant, anti-inflammatory, and cardiometabolic effects in both in vitro and in vivo models [127,128,129,130]. Among these, esterification with SCFAs has shown particular promise in enhancing resveratrol’s therapeutic potential, especially within DOHaD research contexts [26].

#### 5.3.1. Synthesis and Characterization of Resveratrol–SCFA Esters

In our previous work, we synthesized a series of resveratrol–SCFA esters using Steglich esterification, a mild and efficient method commonly used in organic synthesis [131,132]. By reacting resveratrol with 12 different fatty acids (ranging from C3:0 to C22:6), we demonstrated that antioxidant activity is influenced by both chain length and assay type: long-chain esters (e.g., C18:0, C18:1) exhibited higher activity in the DPPH assay, whereas SCFA esters (C3:0–C6:0) were more effective in the ABTS assay [26]. These findings underscore how esterification can modulate the bioactivity of resveratrol depending on the chemical structure and substitution pattern.

Specifically, resveratrol–SCFA esters were synthesized by reacting *trans*-resveratrol with acetic acid, propionic acid, or n-butyric acid, yielding resveratrol acetic acid ester (RAE), resveratrol propionic acid ester (RPE), and resveratrol butyric acid ester (RBE), respectively [26]. The reactions produced mixtures of mono-, di-, and tri-esters. For acetic acid, the distribution of products was as follows: RAE monoester (49.64%), resveratrol (25.34%), and RAE diester (23.40%). When using propionic acid, the resulting compounds included RPE monoester (45.81%), RPE diester (32.80%), and resveratrol (19.91%). With butyric acid, the composition consisted of RBE monoester (47.12%), RBE diester (35.00%), and resveratrol (17.11%). Overall, the yields from the Steglich esterification (73.04–82.12%) were significantly higher compared to those obtained using traditional methods (37.7–74%) [26].

#### 5.3.2. Antioxidant Activity of Resveratrol–SCFA Esters

The antioxidant activity of resveratrol–SCFA esters was assessed using corn oil oxidation, LDL oxidation, and DNA protection assays [133,134,135]. Among the esters, resveratrol acetic acid ester (RAE) exhibited the strongest antioxidant effect in the corn oil model, as indicated by the lowest p-anisidine value (10.71), reflecting superior lipid protection. In contrast, resveratrol RPE showed the highest inhibition (79.0%) of Cu^2+^-induced LDL oxidation, suggesting greater efficacy in metal-catalyzed oxidative systems. These results are consistent with previous studies highlighting distinct antioxidant profiles among resveratrol ester derivatives [136]. Furthermore, all resveratrol–SCFA esters significantly reduced hydrogen peroxide-induced DNA damage, underscoring their role as effective radical scavengers. Notably, resveratrol–SCFA esters exhibited stronger hydrogen peroxide scavenging activity than resveratrol itself, emphasizing the potential of esterification to enhance antioxidant capacity.

#### 5.3.3. Prevention of CKM Syndrome by Resveratrol–SCFA Esters

RBEs have been studied across various animal models for their protective effects against CKM conditions. In a BPA exposure model, perinatal BPA led to weight gain, lipid accumulation, hyperlipidemia, and gut dysbiosis in 50-day-old female offspring, all of which were mitigated by RBE supplementation (30 mg/kg/day) [137]. In male offspring, RBEs also conferred protection against oxidative stress, gut microbiota alterations, and liver damage induced by BPA [133]. These benefits were associated with upregulation of hepatic antioxidant genes (Nrf2, HO-1, SOD, CAT) and reduced liver inflammation. RBEs also promoted gut health by increasing beneficial bacteria such as S24-7 and *Adlercreutzia*, along with elevating SCFA levels [138].

In another study, the protective effect of RBEs was examined in a maternal di-2-ethylhexylphthalate (DEHP) exposure model [139]. Perinatal DEHP exposure led to hypertension and weight gain in 12-week-old male offspring. High-dose RBE (6.67 mg/kg/day) prevented both outcomes, whereas low-dose RBE (3.33 mg/kg/day) failed to sustain the antihypertensive effect by 12 weeks. Resveratrol (6.67 mg/kg/day) was as effective as high-dose RBE in preventing hypertension but did not affect body weight. The benefits of RBE were linked to reduced oxidative stress, improved gut microbiota composition, and restoration of the gut–kidney axis via modulation of butyrate and SCFA receptors [139].

RBEs consist of mono-, di-, and tri-butanoyl resveratrol esters [25]. Among them, two key compounds—3,4′-di-O-butanoylresveratrol (ED2) and 3-O-butanoylresveratrol (ED4)—were isolated and further evaluated due to their superior antioxidant activity [25]. In a maternal high-fructose diet model, ED2 or ED4 (25 mg/L in drinking water) was administered throughout gestation and lactation [140]. The HF diet induced hypertension in male offspring, which was significantly alleviated by ED2 or ED4 supplementation. These effects were linked to enhanced antioxidant activity and increased NO bioavailability. ED2 also modulated gut microbiota, increasing *Bifidobacterium* and *Clostridium* while reducing *Angelakisella* and *Christensenella*. ED4 improved SCFA levels and receptor expression while reducing TMAO levels. These findings highlight ED2 and ED4 as promising interventions for preventing high-fructose diet-induced hypertension in offspring.

#### 5.3.4. Formulation, Delivery, and Regulatory Considerations

The successful clinical translation of resveratrol–SCFA esters hinges on optimized formulation and delivery strategies to improve bioavailability and target tissue distribution. Owing to their enhanced lipophilicity relative to parent resveratrol, these esters are well-suited for incorporation into advanced drug delivery systems such as lipid-based nanoparticles, liposomes, or polymeric micelles, which can protect against rapid metabolism and enable sustained release. Oral administration remains the preferred route given resveratrol’s dietary origin; however, novel approaches including enteric coatings or colon-targeted delivery systems may further enhance intestinal absorption and promote beneficial interactions with the gut microbiota.

From a regulatory perspective, despite the promising pharmacological profiles of resveratrol–SCFA esters and other resveratrol-related derivatives, their clinical development requires rigorous evaluation of dose equivalence, safety, and compliance with regulatory standards. Translational dose extrapolation from animal models must consider species-specific differences in metabolism and bioavailability, commonly utilizing allometric scaling to estimate human-equivalent doses [141,142]. While most derivatives have exhibited low toxicity in preclinical studies, potential risks such as hepatic or gastrointestinal adverse effects remain at high or prolonged exposures. Regulatory approval will necessitate comprehensive toxicological assessments aligned with OECD [143] and FDA guidelines [144], including acute, subchronic, and genotoxicity testing. Furthermore, chemical modification of resveratrol may reclassify these esters from dietary supplements to new chemical entities, thereby requiring full investigational new drug (IND) application and evaluation [145]. Consequently, well-designed pharmacokinetic and safety studies are essential to bridge preclinical findings with clinical translation.

## 6. Conclusions and Future Perspectives

CKM syndrome has emerged as a major public health concern, with an estimated 90% of adults in the United States affected [146]. However, the mechanisms by which early-life risk factors contribute to CKM syndrome and effective preventive strategies remain unclear [147]. Although various medications—such as statins, glucagon-like peptide-1 receptor agonists, and sodium–glucose cotransporter-2 inhibitors—are recommended by AHA guidelines for slowing CKD progression and preventing CVD events [1], their roles in developmental programming of CKM syndrome are underexplored and warrant further investigation.

**Figure 1:**
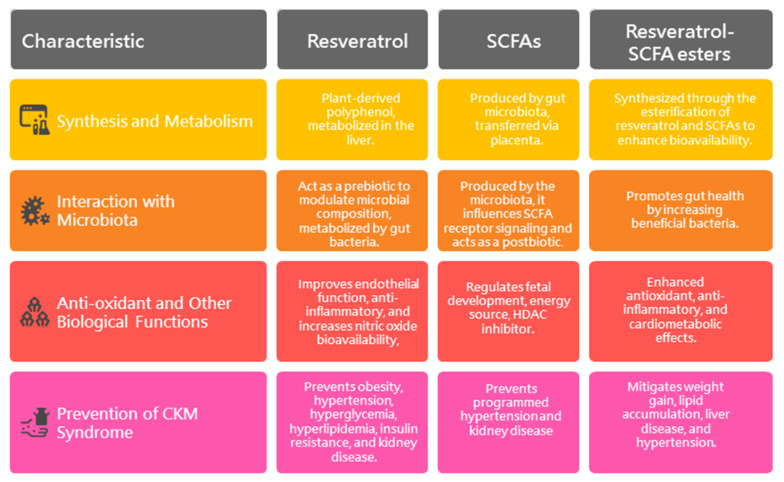
Comparison of the above-mentioned interventions for preventing CKM syndrome.

This review thoroughly examines the effects of resveratrol, SCFAs, and their derivatives during pregnancy and lactation on offspring outcomes, with a focus on CKM syndrome. Their comparisons are summarized and illustrated in Figure 1. It synthesizes current knowledge and highlights emerging opportunities for CKM prevention through interventions targeting oxidative stress and gut microbiota dysbiosis. Given the accumulating evidence that both oxidative stress and gut dysbiosis are key contributors to CKM programming, refining interventions that address both pathways represents a promising strategy to reduce the global burden of CKM syndrome.

To maximize the translational potential of early-life interventions, future efforts should be aligned with existing maternal–child health frameworks and public health nutrition policies. Incorporating evidence-based strategies—such as antioxidant-rich and microbiota-supporting diets—into prenatal care guidelines and early childhood nutrition programs may offer scalable and cost-effective solutions for CKM prevention [148,149]. Breast milk, which naturally provides antioxidants and supports gut microbiota development, represents a promising, holistic approach that aligns with these preventive goals [150]. Furthermore, integration of these approaches into community health systems, maternal education, and national health promotion initiatives could facilitate widespread implementation, particularly in high-risk or underserved populations [151,152]. Such alignment would not only accelerate the translation of preclinical findings into actionable strategies but also support lifespan health equity and the sustainable reduction of CKM-related disease burden.

In the context of resveratrol and SCFAs as early-life interventions for CKM syndrome, population-level variation, sex-specific metabolism, and human cohort validation are critical factors for clinical translation. Population-level variation—including differences in genetics [153], diet [154], and microbiota [155]—can influence individual responses to these compounds, affecting their efficacy and safety across diverse groups. Sex-specific metabolism highlights that males and females may respond differently to resveratrol [156] and SCFAs [157] due to hormonal and metabolic differences, particularly during critical developmental windows. Finally, human cohort validation is essential to confirm findings from animal models in real-world settings [158,159,160], ensuring that early-life interventions are effective and applicable in clinical practice.

Beyond resveratrol, several polyphenols—including quercetin and isoflavones—have demonstrated antioxidant properties [161,162] and modulate gut microbiota [163,164]; yet, none have been evaluated in the context of CKM syndrome with developmental origins. Numerous perinatal antioxidant interventions, such as vitamins C [165] and E [166], L-arginine [167], L-citrulline [168], L-taurine [169], melatonin [170], and N-acetylcysteine [47,171], have shown potential as reprogramming therapies across various CKM phenotypes. Emerging research also links gut microbiota with these amino acids and vitamins, suggesting that their reprogramming effects may involve microbiota modulation [172,173,174].

Interventions targeting the gut microbiota—such as probiotics, prebiotics, and postbiotics—have shown promise in improving CKM-related phenotypes in preclinical models [175,176,177,178,179,180]. Among these, postbiotics have garnered increasing attention due to their ability to beneficially modulate the gut microbiome without the safety concerns associated with live microorganisms [181]. SCFAs, a well-studied class of postbiotics, have demonstrated cardiovascular benefits in animal studies [182]; however, data supporting their efficacy in improving broader CKM outcomes—particularly when administered during the perinatal period—remain limited. Moreover, the mechanistic links between postbiotics, oxidative stress modulation, and long-term CKM risk are not yet fully understood [183].

To bridge the translational gap, future research must conduct rigorous clinical trials to evaluate the safety and efficacy of resveratrol, SCFAs, and related interventions during pregnancy and lactation. These trials should focus on intervention timing, targeting critical developmental windows with well-characterized protocols. Integrating multi-omics approaches—including metabolomics, microbiome, and epigenetic profiling—will be essential for uncovering causal mechanisms and identifying predictive biomarkers. Furthermore, although many natural and synthetic resveratrol derivatives have demonstrated bioactivity in vitro and in animal studies [130], only a limited subset has been evaluated in the context of developmental reprogramming for CKM syndrome. Advancing these candidates into precision prevention strategies, especially in high-risk mother-infant cohorts, is crucial for transforming preclinical evidence into effective human therapies.

**Figure 2:**
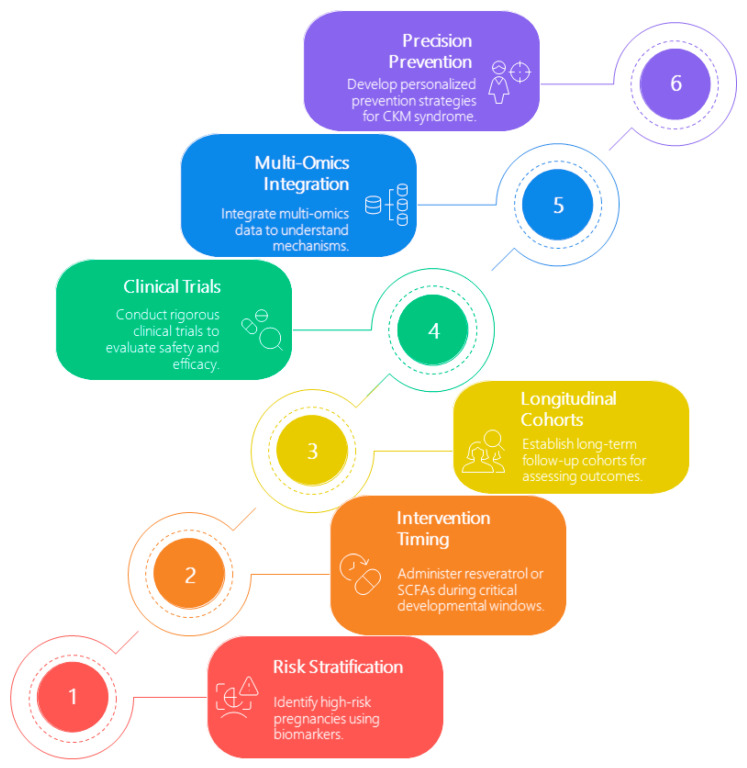
Conceptual framework outlining six key pillars to bridge preclinical research and clinical application for early prevention of CKM syndrome.

To accelerate clinical translation, we propose a framework comprising six key pillars (Figure 2):

1. Risk Stratification—Identify high-risk pregnancies using early-life biomarkers such as maternal microbiota profiles and oxidative stress indicators.
2. Intervention Timing—Administer targeted interventions (e.g., SCFAs) during sensitive developmental periods to optimize impact.
3. Longitudinal Cohorts—Establish long-term follow-up birth cohorts to track CKM-related outcomes.
4. Clinical Trials—Rigorously assess the safety, dosage, and efficacy of candidate interventions in controlled studies.
5. Multi-Omics Integration—Leverage systems biology to elucidate molecular mechanisms and identify therapeutic targets.
6. Precision Prevention—Develop personalized strategies tailored to genetic, microbiota, and sex-specific variations to prevent CKM syndrome.

In conclusion, early-life interventions targeting oxidative stress and gut microbiota offer a novel and potentially transformative approach for CKM prevention. Bridging experimental findings with well-designed human studies could not only reduce CKM-related morbidity and mortality but also inform precision prevention strategies with broad public health impact.

## Figures and Tables

**Table 1:** Animal studies showing that resveratrol prevents offspring CKM syndrome.

Resveratrol Dose	Supplementation Period	Experimental Condition	Animal Model/Sex	Age at Evaluation (Weeks)	Prevented Phenotypes	Ref.
50 mg/L	Gestation + Lactation	Maternal high-fat diet	Wistar rats/M & F	3	Obesity, hyperglycemia, hyperlipidemia	[81]
50 mg/L	Gestation + Lactation	High-fructose diet (maternal + post-weaning)	SD rats/M	12	Hypertension	[61]
50 mg/L	Gestation + Lactation	Maternal CKD	SD rats/M	12	Hypertension, kidney disease	[82]
50 mg/L	Gestation + Lactation	Maternal ADMA + TMAO	SD rats/M	12	Hypertension	[83]
50 mg/L	Gestation + Lactation	Maternal TCDD exposure	SD rats/M	12	Hypertension	[84]
50 mg/L	Gestation + Lactation	High-fat diet (maternal + post-weaning)	SD rats/M	16	Obesity, hyperlipidemia, hypertension	[85,86]
50 mg/L	Gestation + Lactation	Maternal L-NAME + high-fat diet	SD rats/M	16	Hypertension	[87]
50 mg/L	Gestation + Lactation	Maternal TCDD + dexamethasone	SD rats/M	16	Hypertension	[88]
50 mg/L	Gestation + Lactation	Bisphenol A + high-fat diet	SD rats/M	16	Hypertension	[89]
25 mg/kg/day	Gestation only	Maternal protein restriction	Wistar rats/M & F	16	Obesity, insulin resistance	[90]
4 g/kg in diet	Gestation + Lactation	Maternal hypertension	SHR/M & F	20	Hypertension	[91]
0.2% in diet	Gestation + Lactation	High-fat diet (maternal + post-weaning)	C57BL/6J mice/M	14	Obesity, hyperlipidemia	[92]

ADMA = asymmetric dimethylarginine; TMAO = trimethylamine-N-oxide; TCDD = 2,3,7,8-tetrachlorodibenzo-p-dioxin; SD rats = Sprague–Dawley rats; SHR = spontaneously hypertensive rat; M = male; F = female.

**Table 2:** Animal studies representing SCFAs prevent offspring CKM syndrome.

SCFA	Supplementation Period	Experimental Condition	Animal Model/Sex	Age at Evaluation (Weeks)	Prevented Phenotypes	Ref.
Magnesium acetate 200 mmol/L	Gestation + Lactation	Maternal high-fructose diet	SD rats/M	12	Hypertension	[114]
Magnesium acetate 200 mmol/L	Gestation + Lactation	Maternal minocycline exposure	SD rats/M	12	Hypertension	[115]
Sodium butyrate 400 mmol/L	Gestation + Lactation	Maternal tryptophan-free diet	SD rats/M	12	Hypertension	[116]
Sodium butyrate 400 mmol/L	Gestation + Lactation	Maternal high-fructose diet	SD rats/M	12	Hypertension	[117]
Propionate 200 mmol/L	Gestation + Lactation	Maternal high-fructose diet	SD rats/M	12	Hypertension	[117]
Propionate 200 mmol/L	Gestation + Lactation	Maternal CKD	SD rats/M	12	Hypertension, kidney disease	[118]

SD rats = Sprague–Dawley rats; M = male; CKD = chronic kidney disease.

## Data Availability

Data are contained within the article.
